# Depth-Related Curing Potential of Ormocer- and Dimethacrylate-Based Bulk-Fill Composites

**DOI:** 10.3390/ma14226753

**Published:** 2021-11-09

**Authors:** Ramona S. Oltramare, Reto Odermatt, Phoebe Burrer, Thomas Attin, Tobias T. Tauböck

**Affiliations:** 1 Clinic of Conservative and Preventive Dentistry, Center for Dental Medicine, University of Zurich, 8032 Zurich, Switzerland; phoebe.burrer@zzm.uzh.ch (P.B.); thomas.attin@zzm.uzh.ch (T.A.); tobias.tauboeck@zzm.uzh.ch (T.T.T.); 2 Department of Pediatric Oral Health and Orthodontics, University Center for Dental Medicine Basel UZB, University of Basel, 4058 Basel, Switzerland; r.odermatt@unibas.ch

**Keywords:** bulk-fill resin composite, degree of conversion, ormocer-based bulk-fill composite, polymerization

## Abstract

The aim of this in vitro study was to investigate the degree of C=C double bond conversion of high-viscosity dimethacrylate- or ormocer-based bulk-fill composites as a function of measurement depth. Four bulk-fill composites (Tetric EvoCeram Bulk Fill, x-tra fil, SonicFill, and Bulk Ormocer) and the conventional nanohybrid composite Tetric EvoCeram were applied in standardized Class II cavities (*n* = 6 per group) and photoactivated for 20 s at 1350 mW/cm^2^. The degree of conversion of the composites was assessed using Fourier-transform infrared spectroscopy at seven measurement depths (0.15, 1, 2, 3, 4, 5, 6 mm). Data were analyzed using repeated measures ANOVA and one-way ANOVA with Bonferroni post-hoc tests (α = 0.05). The investigated bulk-fill composites showed at least 80% of their maximum degree of conversion (80% DC_max_) up to a measuring depth of at least 4 mm. Tetric EvoCeram Bulk Fill and Bulk Ormocer achieved more than 80% DC_max_ up to a measuring depth of 5 mm, x-tra fil up to 6 mm. The conventional nanohybrid composite Tetric EvoCeram achieved more than 80% DC_max_ up to 3 mm. In contrast to the conventional composite, the investigated ormocer- and dimethacrylate-based bulk-fill composites can be photo-polymerized in thick layers of up to at least 4 mm with regard to their degree of C=C double bond conversion.

## 1. Introduction

The application of resin-based composites, which are widely used as dental restoration materials [1], offers a wide range of indications, but is quite challenging. Not only the pretreatment with an appropriate adhesive system shows a certain technique sensitivity [2]. The actual application of the composite material also needs to be performed meticulously, since a maximum layer thickness of 2 mm should usually not be exceeded [3]. On the one hand, this allows adequate polymerization of the resin matrix during light activation; on the other hand, it minimizes polymerization-induced shrinkage stresses. For large cavities, this means that several individual increments have to be layered on top of each other, which is not only time-consuming, but can also lead to air inclusions and contamination [4].

To simplify application and make it more time efficient, bulk-fill composites have been developed. According to the manufacturers, bulk-fill composites can be applied and photoactivated at a layer thickness of 4–5 mm without compromising polymerization in deep portions of the materials [5]. By modifying the material composition, these higher incremental layer thicknesses can be achieved. In general, bulk-fill composites show higher translucency than conventional composites [5,6] due to a lower filler loading and/or larger incorporated filler particles, which reduces light scattering at filler–matrix interfaces and ensures that more light can penetrate into deeper layers [6,7]. Besides modification of the translucency, the photo-initiator has also been adapted in some bulk-fill materials [8,9]. Instead of the classic camphorquinone (CQ), Tetric EvoCeram Bulk Fill contains Ivocerin as an additional initiator system. This germanium-based initiator system exhibits higher light absorption in the range between 400 and 450 nm compared to CQ, which results in higher light-curing activity. In addition, this initiator is more efficient than CQ because it forms at least two radicals that can initiate radical polymerization, in contrast to CQ, which only forms one radical [10].

Organically modified ceramics, so-called ormocers, represent a newly developed material class. In an attempt to overcome the disadvantages of polymerization shrinkage, ormocers consist not only of an organic but also of an inorganic network. This network is supposed to embed the monomers better and lead to a lower monomer release compared to conventional composites [11]. Ormocer-based bulk-fill composites have not yet been extensively researched, particularly regarding their curing potential in deep material layers. Furthermore, the majority of studies investigated the polymerization behavior of composite materials in Teflon or metal molds [12,13,14,15]. Since light scattering differs between Teflon or metal and tooth surfaces, these results cannot be transferred unconditionally to the clinical situation [16]. Clinical situations can be better simulated if tooth cavities are used in the investigations.

The aim of this in vitro study was to investigate the degree of C=C double bond conversion of ormocer- and dimethacrylate-based bulk-fill composites applied in tooth cavities as a function of composite layer thickness. Composite layer thicknesses up to 6 mm were investigated. The null hypothesis tested was that there are no significant differences between conventional and bulk-fill composites in terms of the depth-related degree of conversion.

## 2. Materials and Methods

### 2.1. Specimen Preparation

Fifteen unrestored, caries-free human molars with completed root growth were used. The teeth were extracted for therapeutic reasons, with the patients’ written consent that they could be used for research purposes. Since the teeth were irreversibly anonymized after extraction, the research was compatible with the use of anonymized biological material and thus exempt from getting ethical approval (Federal Act on Research involving Human Beings (Human Research Act; article 2, paragraph 2)).

**Figure 1:**
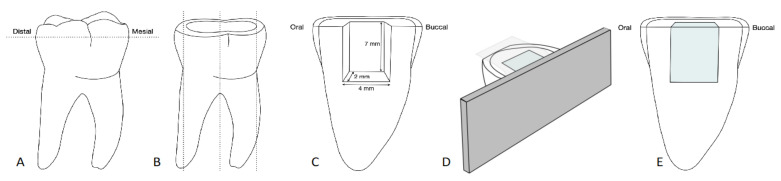
Schematic representation of specimen preparation: (**A**) Reduction of cusps to obtain a flat surface; (**B**) three parallel cuts to obtain tooth halves; (**C**) preparation of the standardized cavity in the tooth half; (**D**) composite application using a Mylar strip and a metal plate; (**E**) final specimen with composite filling.

The cleaned teeth were fixed to a specimen carrier (SEM Mounts; Baltec, Balzers, Liechtenstein) with a self-curing acrylic resin (Paladur; Heraeus Kulzer, Hanau, Germany). Then, the cusps of the teeth were reduced with 120-grit silicon carbide paper (Struers, Bellerup, Denmark) using a polishing machine (Planopol-2; Struers, Bellerup, Denmark) under water cooling until a flat occlusal surface was obtained (Figure 1A). Three parallel bucco-oral cuts were then made with a diamond saw (Isomet; Buehler, Lake Bluff, IL, USA) under water cooling at each tooth crown to obtain two tooth halves (Figure 1B).

### 2.2. Restoration

**Figure 2:**
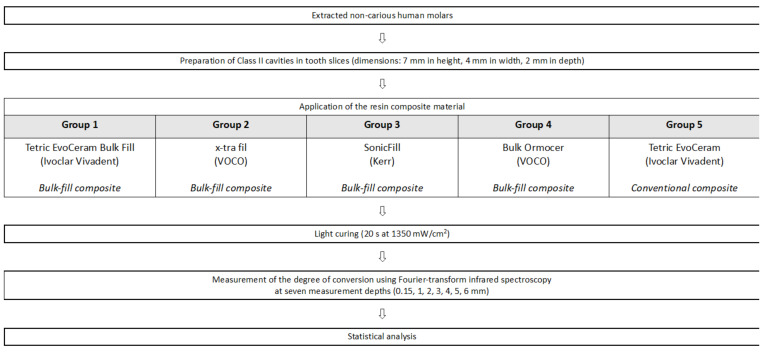
Experimental protocol.

Using a cylindrical 80-µm diamond bur (Universal Prep Set; Intensiv SA, Montagnola, Switzerland), standardized cavities of 7 mm height, 4 mm width and 2 mm depth were prepared in the tooth discs under water cooling (Figure 1C). The dimensions of the cavities were checked by using a periodontal probe. The tooth cavities were randomly divided into five groups (*n* = 6 per group) and filled in one increment according to their group with one of the four bulk-fill composites or the conventional composite, as detailed in Figure 2. Table 1 shows the composition of the composite materials.

The filled cavities were laterally covered with a Mylar Strip (Kerr, Orange, CA, USA) and pressed against a metal plate. The Mylar Strip was then pressed occlusally over the cavity opening and the excess material was removed (Figure 1D). The composite material was light-cured from the occlusal surface by holding the LED curing unit (Celalux 3; VOCO, Cuxhaven, Germany) in direct contact with the Mylar Strip while centered on top of the cavity. Light curing was performed for 20 s at an irradiance of 1350 mW/cm^2^, which was monitored using a PM2 thermopile sensor and a calibrated FieldMaxII-TO power meter (Coherent, Santa Clara, CA, USA). After light curing, the metal plate and Mylar strip were removed. The specimens (Figure 1E) were stored for 96 h in the dark at room temperature (22 ± 1 °C).

### 2.3. Degree of Conversion Analysis

The degree of conversion (DC) was quantified using a Fourier-transform infrared spectrometer (Lumos; Bruker Corporation, Billerica, MA, USA) equipped with an attenuated total reflectance (ATR) device equipped with a germanium crystal. The contact area of this integrated ATR crystal had a diameter of 100 µm, and the best spatial resolution for the measurement with the ATR crystal was 1.25 µm. Measurements were made fully automatically after establishing the measurement points at the different depths (0.15, 1, 2, 3, 4, 5, 6 mm). Three measurements were made at each depth. The infrared spectra were recorded in a wavenumber range from 4000–300 cm^−1^ with 32 scans at a resolution of 4 cm^−1^.

The degree of conversion (DC) was calculated using the following equation [17]:

(1) $$\mathrm{DC}\;\left(\%\right)=\left(1-\frac{Rpolymerized}{Runpolymerized}\right)\times 100$$

*R* corresponds to the ratio of the absorption intensities of the peak areas at 1638 and 1608 cm^−1^ in the spectra of the dimethacrylate-based composites (Tetric EvoCeram Bulk Fill, x-tra fil, Sonic Fill, Tetric EvoCeram), or the ratio of the absorption intensities of the peak areas at 1638 and 1592 cm^−1^ in the spectra of the ormocer-based composite (Bulk Ormocer).

### 2.4. Statistical Analysis

Data were analyzed using SPSS (version 20; IBM, Armonk, NY, USA). Normal distribution of the data was verified using the Kolmogorov–Smirnov test. DC values at different depths (within a material) were compared using repeated measures ANOVA together with Greenhouse–Geisser correction and matching contrast. DC values of different materials (within a measurement depth) were compared using one-way ANOVA followed by Bonferroni post-hoc tests. For all statistical analyses, the level of significance was set at α = 0.05.

## 3. Results

The results of the degree of conversion (DC) measurements are presented in Table 2. All bulk-fill composites achieved at least 80% of their maximum DC (80% DC_max_) up to a depth of at least 4 mm. If the value 80% DC_max_ was exceeded, this was interpreted as adequate polymerization [18].

Tetric EvoCeram Bulk Fill achieved the lowest DC among the tested materials (46.4%) at the near surface (0.15 mm). At least 80% DC_max_ was achieved up to a depth of 5 mm, from where a significant decrease in the DC was observed compared to the near surface.

SonicFill achieved the highest maximum DC of all materials (79.5%) and reached at least 80% DC_max_ up to a depth of 4 mm. However, the DC already decreased significantly at depths of 2 mm and beyond, when compared to the near surface.

x-tra fil achieved at least 80% DC_max_ up to 6 mm depth and Bulk Ormocer up to 5 mm depth. Corresponding significant decreases in the DC relative to the near surface were observed from 5 mm and 6 mm depth, respectively.

The conventional composite Tetric EvoCeram reached at least 80% DC_max_ up to a depth of 3 mm, from where a significant DC decrease was observed relative to the near surface. At 4 mm depth and beyond, Tetric EvoCeram attained the significantly lowest DC of all materials investigated.

SonicFill and Tetric EvoCeram achieved their maximum DC at the near surface (0.15 mm), while for Tetric EvoCeram Bulk Fill, x-tra fil, and Bulk Ormocer, their maximum DC was reached at a depth of 1 mm.

## 4. Discussion

In this in vitro study, the degree of conversion (DC) of the ormocer- and dimethacrylate-based bulk-fill composites was significantly higher at depths of 4 mm and beyond, when compared to a conventional composite. Thus, the null hypothesis was rejected.

To determine DC at different layer depths, many studies use Teflon or metal as mold materials [12,13,14,15,19]. However, depending on the material used, the light transmission and reflection may vary [16]. Thus, the results of the investigations are significantly dependent on the material of the molds and cannot be extrapolated uniformly to tooth cavities. It was shown that more light is available at the bottom of a filled tooth cavity than at the bottom of a filled metal cavity [16]. To apply the results of this study more reliably to the clinical situation, human molars were used in the current experimental set-up.

In the present study, Tetric EvoCeram Bulk Fill achieved at least 80% of its maximum DC (80% DC_max_) up to a depth of 5 mm. However, the near surface showed the lowest value (46.4%) of all composites examined and the measured values did not differ significantly from the conventional composite (Tetric EvoCeram) within the respective depth up to a depth of 3 mm. In contrast to our results, Garoushi et al. [20] reported that Tetric EvoCeram Bulk Fill could not be used at a layer thickness of 4 mm or more. The different results could be explained by the different mold materials (Teflon compared to tooth) on the one hand, and by different evaluation methods on the other hand. Further studies, however, confirmed good polymerization behavior of Tetric EvoCeram Bulk Fill [18,21], which might be attributed to the fact that the initiator (Ivocerin) in this composite ensures efficient curing [10].

SonicFill achieved the significantly highest DC of all materials investigated up to a depth of 4 mm. Only at a depth of 6 mm did x-tra fil and Bulk Ormocer show higher values (50.9% and 48.7%, respectively) than SonicFill (42.0%). Other studies have confirmed the high maximum DC of SonicFill [18,20], which might be due to the used monomer system. The DC decreases with increasing Bis-GMA concentration, and increases with increasing TEGDMA concentration [22]. A high TEGDMA/Bis-GMA ratio could thus be one reason for the observed high maximum DC of SonicFill. Another reason might be the high photoinitiator content of SonicFill, as stated by the manufacturer. On the other hand, SonicFill was the only composite examined in this study that did not meet the manufacturer’s stated specifications in terms of depth of cure. Instead of the stated 5 mm, SonicFill achieved 80% DC_max_ only up to a depth of 4 mm. This result was confirmed in a study by Goracci et al. [23] and might be explained by the lower translucency of SonicFill compared to other bulk-fill composites [6]. Low translucency results in fewer photons penetrating into deep material layers during light polymerization, where fewer photoinitiators can then be activated [18]. In addition, SonicFill has more irregularly shaped fillers compared to other bulk-fill composites, which further reduces light transmission through the material [6]. It should be noted, however, that even if the 80% of DC_max_ was not reached at a depth of 4 mm, SonicFill achieved a higher value at this depth with a DC of 69.8% than all other composites investigated at the near surface.

X-tra fil was the only material to reach more than 80% DC_max_ up to a depth of 6 mm. With a DC of 50.9%, x-tra fil reached the highest value at this depth of all materials investigated. The high depth of cure of x-tra fil, which has also been reported in other studies [18,20], might be explained by the large filler size (up to 10 µm) of the material. As a result, for the same filler content, the total filler surface area decreases, which reduces light scattering at filler–matrix interfaces, allowing more light to penetrate into deeper layers [7].

The experimental ormocer-based composite Bulk Ormocer showed more than 80% DC_max_ up to a depth of 5 mm, which confirms its suitability as a bulk-fill material. At measurement depths of 5–6 mm, it was amongst the materials with the highest C=C double bond conversion. Along with x-tra fil (50.9%), Bulk Ormocer had a higher DC (48.7%) at a depth of 6 mm than Tetric EvoCeram Bulk Fill and Tetric EvoCeram at the near surface (46.4% and 46.8%, respectively). In addition to high depth of cure, low shrinkage stress development has been reported for the ormocer-based bulk-fill composite (meanwhile approved for the market under Admira Fusion x-tra) [13]. Bulk Ormocer contains inorganic-organic copolymers instead of classical monomers. Together with the lower organic resin amount, this ensures reduced polymerization shrinkage and stress development [24,25,26]. As a result of its low shrinkage stress formation, low cuspal deflection [27] and a favorable high margin integrity have been found for Admira Fusion x-tra [28]. Furthermore, the reduced polymerization rate of the ormocer matrix gives the polymer network forming more time to reorganize. Newly emerged shrinkage forces can thus be relieved by viscous flow and molecular relaxation in the early curing phase [13,28,29].

The conventional composite (Tetric EvoCeram) achieved more than 80% DC_max_ up to a layer thickness of 3 mm, which exceeds the maximum layer thickness specified by the manufacturer (1.5–2 mm). The conventional composite thus seems to tolerate an application beyond the recommended maximum layer thickness. However, it should not be used in the same range of indications as the tested bulk-fill composites due to insufficient polymerization in deeper material layers.

The 80% DC_max_ criterion is often chosen to estimate the quality of polymerization at deeper layer thicknesses. However, this criterion also has certain disadvantages, as it only allows a relative estimate of the extent of polymerization. Since the DC_max_ varied greatly between the materials investigated, the 80% DC_max_ also showed a corresponding variation. Until now, no threshold value of DC has been established which defines adequate curing [30]. However, it was suggested that a DC of at least 55% should be achieved, as values below correlate with reduced mechanical stability [31,32]. According to this consideration, the DC values of Tetric EvoCeram Bulk Fil (46.4%) and Tetric EvoCeram (46.8%) would already be inadequate at the near surface. In contrast, x-tra fil, SonicFill and Bulk Ormocer exceeded the suggested DC of 55% not only at the near surface, but also at the respective specified maximum layer thicknesses of 4 mm (x-tra fil and Bulk Ormocer) or 5 mm (SonicFill).

## 5. Conclusions

The examined bulk-fill composites can be applied at layer thicknesses of up to 4 mm (SonicFill) or more (Tetric EvoCeram Bulk Fill, x-tra fil, Bulk Ormocer) based on the 80% DC_max_ criterion. The conventional composite under investigation exceeded the depth of cure specified by the manufacturer based on the 80% DC_max_ criterion but should not be used at similar layer thickness as the bulk-fill composites. SonicFill showed the highest absolute DC values up to a depth of 5 mm with values exceeding 55% at this depth. Tetric EvoCeram Bulk Fil and Tetric EvoCeram, on the other hand, did not attain the suggested 55% even at the near surface.

## Figures and Tables

**Table 1:** Manufacturers’ information on the composite materials used in this study.

Composite	Manufacturer	Composition	Filler Size (µm)	Filler Content (wt %/vol %)	LOT
Tetric EvoCeram Bulk Fil	Ivoclar Vivadent, Schaan, Liechtenstein	**Matrix:** Bis-GMA ^1^, Bis-EMA ^2^, UDMA ^3^ **Filler:** Ba-Al-Si-glass, YbF_3_, spherical mixed oxide, PPF ^4^ (monomer, glass filler and ytterbium fluoride)	0.04–3 (Mean value: 0.55)	81/61	T28064
x-tra fil	VOCO, Cuxhaven, Deutschland	**Matrix:** Bis-GMA ^1^, UDMA ^3^, TEGDMA ^5^ **Filler:** Ba-B-Al-Si-glass	0.05–10 (Mean value: 3)	86/70	1438594
SonicFill	Kerr, Orange, CA, USA	**Matrix:** Bis-GMA ^1^, Bis-EMA ^2^, TEGDMA ^5^ **Filler:** Ba-B-Al-Si-glass, SiO_2_	Not indicated	83.5/66	5338301
Bulk Ormocer	VOCO, Cuxhaven, Deutschland	**Matrix:** Ormocer-matrix **Filler:** SiO_2_, glass ceramics	0.02–3	84/69	1441426
Tetric EvoCeram	Ivoclar Vivadent, Schaan, Liechtenstein	**Matrix:** Bis-GMA ^1^, Bis-EMA ^2^, UDMA ^3^ **Filler:** Ba-Al-Si-glass, YbF_3_, spherical mixed oxide, PPF ^4^ (monomer, glass filler and ytterbium fluoride)	0.04–3 (Mean value: 0.55)	76/55	T37817

^1^ Bis-GMA: bisphenol-A-glycidyldimethacrylate; ^2^ Bis-EMA: ethoxylated bisphenol-A-dimethacrylate; ^3^ UDMA: urethane dimethacrylate; ^4^ PPF: prepolymer filler; ^5^ TEGDMA: triethylene glycol dimethacrylate.

**Table 2:** Mean (±standard deviation) degree of conversion (DC, in %) of the tested composites at different measurement depths.

Group	Material	Measuring Depth (mm)							80% DC_max *_
		0.15	1	2	3	4	5	6	
1	Tetric EvoCeram Bulk Fil	46.4 (4.5) **ABCc**	48.8 (3.0) **Ac**	47.8 (3.0) **ABc**	44.3 (1.2) **BCc**	43.3 (2.0) **Cc**	39.6 (1.4) **Db**	32.3 (3.6) **Ec**	39.0
2	x-tra fil	57.6 (4.0) **ABb**	61.0 (2.7) **Ab**	59.2 (3.0) **ABb**	59.3 (1.9) **Ab**	56.8 (1.7) **Bb**	53.9 (2.5) **Ca**	50.9 (1.5) **Da**	48.8
3	SonicFill	79.5 (1.8) **Aa**	79.2 (4.3) **Aa**	76.0 (3.8) **Ba**	75.5 (1.7) **Ba**	69.8 (2.2) **Ca**	57.5 (2.8) **Da**	42.0 (4.9) **Eb**	63.6
4	Bulk Ormocer	61.5 (3.3) **ABCb**	63.3 (2.3) **Ab**	61.4 (4.7) **ABb**	61.3 (2.9) **Ab**	57.3 (3.0) **BCb**	55.5 (3.6) **Ca**	48.7 (3.0) **Dab**	50.7
5	Tetric EvoCeram	46.8 (3.5) **Ac**	45.4 (2.6) **Ac**	45.4 (4.3) **ABc**	43.2 (2.6) **Bc**	36.8 (4.7) **Cd**	30.3 (3.4) **Dc**	20.0 (5.1) **Ed**	37.4

* 80% of the maximum DC. Different upper case letters indicate significant differences in DC between measurement depths within the same material (*p* < 0.05). Different lowercase letters indicate significant differences in DC between materials at the same measurement depth (*p* < 0.05).

## Data Availability

The data presented in this study are available on request from the corresponding author.

## References

[1] Yadav R., Kumar M. (2019). Dental restorative composite materials: A review. J. Oral Biosci..

[2] Cardoso M.V., de Almeida Neves A., Mine A., Coutinho E., Van Landuyt K., De Munck J., Van Meerbeek B. (2011). Current aspects on bonding effectiveness and stability in adhesive dentistry. Aust. Dent. J..

[3] Van Dijken J.W.V., Pallesen U. (2016). Posterior bulk-filled resin composite restorations: A 5-year randomized controlled clinical study. J. Dent..

[4] Park J., Chang J., Ferracane J., Lee I.B. (2008). How should composite be layered to reduce shrinkage stress: Incremental or bulk filling. Dent. Mater..

[5] Van Ende A., De Munck J., Lise D.P., Van Meerbeek B. (2017). Bulk-fill composites: A review of the current literature. J. Adhes. Dent..

[6] Bucuta S., Ilie N. (2014). Light transmittance and micro-mechanical properties of bulk fill vs. conventional resin based composites. Clin. Oral Investig..

[7] Ilie N., Bucuta S., Draenert M. (2013). Bulk-fill resin-based composites: An in vitro assessment of their mechanical performance. Oper. Dent..

[8] Par M., Marovic D., Attin T., Tarle Z., Tauböck T.T. (2020). Effect of rapid high-intensity light-curing on polymerization shrinkage properties of conventional and bulk-fill composites. J. Dent..

[9] Par M., Marovic D., Attin T., Tarle Z., Tauböck T.T. (2020). The effect of rapid high-intensity light-curing on micromechanical properties of bulk-fill and conventional resin composites. Sci. Rep..

[10] Moszner N., Fischer U.K., Ganster B., Liska R., Rheinberger V. (2008). Benzoyl germanium derivatives as novel visible light photoinitiators for dental materials. Dent. Mater..

[11] Polydorou O., König A., Hellwig E., Kümmerer K. (2009). Long-term release of monomers from modern dental-composite materials. Eur. J. Oral Sci..

[12] Dieckmann P., Mohn D., Zehnder M., Attin T., Tauböck T.T. (2019). Light transmittance and polymerization of bulk-fill composite materials doped with bioactive micro-fillers. Materials.

[13] Tauböck T.T., Jäger F., Attin T. (2019). Polymerization shrinkage and shrinkage force kinetics of high- and low-viscosity dimethacrylate- and ormocer-based bulk-fill resin composites. Odontology.

[14] Tauböck T.T., Marovic D., Zeljezic D., Steingruber A.D., Attin T., Tarle Z. (2017). Genotoxic potential of dental bulk-fill resin composites. Dent. Mater..

[15] Yu P., Yap A., Wang X.Y. (2017). Degree of conversion and polymerization shrinkage of bulk-fill resin-based composites. Oper. Dent..

[16] Brandt W.C., Neves A.C.C., de Souza-Junior E.J.C., Sinhoreti M.A.C. (2013). Influence of photoactivation method and mold for restoration on the Knoop hardness of resin composite restorations. Lasers Med. Sci..

[17] Rueggeberg F.A., Hashinger D.T., Fairhurst C.W. (1990). Calibration of FTIR conversion analysis of contemporary dental resin composites. Dent. Mater..

[18] Tarle Z., Attin T., Marovic D., Andermatt L., Ristic M., Tauböck T.T. (2015). Influence of irradiation time on subsurface degree of conversion and microhardness of high-viscosity bulk-fill resin composites. Clin. Oral Investig..

[19] Par M., Spanovic N., Mohn D., Attin T., Tauböck T.T., Tarle Z. (2020). Curing potential of experimental resin composites filled with bioactive glass: A comparison between Bis-EMA and UDMA based resin systems. Dent. Mater..

[20] Garoushi S., Vallittu P., Shinya A., Lassila L. (2016). Influence of increment thickness on light transmission, degree of conversion and micro hardness of bulk fill composites. Odontology.

[21] Zorzin J., Maier E., Harre S., Fey T., Belli R., Lohbauer U., Petschelt A., Taschner M. (2015). Bulk-fill resin composites: Polymerization properties and extended light curing. Dent. Mater..

[22] Amirouche-Korichi A., Mouzali M., Watts D.C. (2009). Effects of monomer ratios and highly radiopaque fillers on degree of conversion and shrinkage-strain of dental resin composites. Dent. Mater..

[23] Goracci C., Cadenaro M., Fontanive L., Giangrosso G., Juloski J., Vichi A., Ferrari M. (2014). Polymerization efficiency and flexural strength of low-stress restorative composites. Dent. Mater..

[24] Ilie N. (2017). Impact of light transmittance mode on polymerisation kinetics in bulk-fill resin-based composites. J. Dent..

[25] Wolter H., Storch W., Ott H. (1994). New inorganic/organic copolymers (Ormocer^®^ s) for dental applications. Mater. Res. Soc. Symp. Proc..

[26] Pick B., Pelka M., Belli R., Braga R.R., Lohbauer U. (2011). Tailoring of physical properties in highly filled experimental nanohybrid resin composites. Dent. Mater..

[27] Yarmohamadi E., Jahromi P.R., Akbarzadeh M. (2018). Comparison of cuspal deflection and microleakage of premolar teeth restored with three restorative materials. J. Contemp. Dent. Pract..

[28] Paganini A., Attin T., Tauböck T.T. (2020). Margin integrity of bulk-fill composite restorations in primary teeth. Materials.

[29] Tauböck T.T., Bortolotto T., Buchalla W., Attin T., Krejci I. (2010). Influence of light-curing protocols on polymerization shrinkage and shrinkage force of a dual-cured core build-up resin composite. Eur. J. Oral Sci..

[30] Alshali R.Z., Silikas N., Satterthwaite J.D. (2013). Degree of conversion of bulk-fill compared to conventional resin-composites at two time intervals. Dent. Mater..

[31] Ferracane J.L., Mitchem J.C., Condon J.R., Todd R. (1997). Wear and marginal breakdown of composites with various degrees of cure. J. Dent. Res..

[32] Silikas N., Eliades G., Watts D.C. (2000). Light intensity effects on resin-composite degree of conversion and shrinkage strain. Dent. Mater..

[33] Odermatt R. (2016). Polymerisationsverhalten hochvisköser dimethacrylat- und ormocerbasierter Bulk-Fill-Komposite in Abhängigkeit von der Schichtstärke. Master’s Thesis.

